# Global burden of heart failure due to cardiovascular diseases, 1990–2021 and predictions to 2035 based on GBD 2021

**DOI:** 10.1093/eschf/xvag038

**Published:** 2026-01-23

**Authors:** Shuang Liu, Chen Zhang, Shizhong Cheng, Xuejin Chen, Qingdui Zhang, Haoran Li, Chengmeng Zhang, Lili Wang, Hao Miao, Qiang Zhou, Lele Wang, Ji Hao, Chunmei Qi, Xiangjie Liu

**Affiliations:** Department of Cardiology, The Second Affiliated Hospital of Xuzhou Medical University, 32 Meijian Road, Quanshan District, Xuzhou City, Jiangsu Province 221000, China; Department of Geriatrics, Liyuan Hospital, Tongji Medical College, Huazhong University of Science and Technology, Wuhan 430000, China; Department of Cardiology, The Second Affiliated Hospital of Xuzhou Medical University, 32 Meijian Road, Quanshan District, Xuzhou City, Jiangsu Province 221000, China; Department of Cardiology, The Second Affiliated Hospital of Xuzhou Medical University, 32 Meijian Road, Quanshan District, Xuzhou City, Jiangsu Province 221000, China; Department of Cardiology, The Second Affiliated Hospital of Xuzhou Medical University, 32 Meijian Road, Quanshan District, Xuzhou City, Jiangsu Province 221000, China; Department of Cardiology, The Second Affiliated Hospital of Xuzhou Medical University, 32 Meijian Road, Quanshan District, Xuzhou City, Jiangsu Province 221000, China; Department of Cardiology, The Second Affiliated Hospital of Xuzhou Medical University, 32 Meijian Road, Quanshan District, Xuzhou City, Jiangsu Province 221000, China; Department of Cardiology, The Second Affiliated Hospital of Xuzhou Medical University, 32 Meijian Road, Quanshan District, Xuzhou City, Jiangsu Province 221000, China; Department of Cardiology, The Second Affiliated Hospital of Xuzhou Medical University, 32 Meijian Road, Quanshan District, Xuzhou City, Jiangsu Province 221000, China; Department of Cardiology, The Second Affiliated Hospital of Xuzhou Medical University, 32 Meijian Road, Quanshan District, Xuzhou City, Jiangsu Province 221000, China; Department of Cardiology, The Second Affiliated Hospital of Xuzhou Medical University, 32 Meijian Road, Quanshan District, Xuzhou City, Jiangsu Province 221000, China; Department of Cardiology, The Second Affiliated Hospital of Xuzhou Medical University, 32 Meijian Road, Quanshan District, Xuzhou City, Jiangsu Province 221000, China; Department of Cardiology, The Second Affiliated Hospital of Xuzhou Medical University, 32 Meijian Road, Quanshan District, Xuzhou City, Jiangsu Province 221000, China; Department of Geriatrics, Liyuan Hospital, Tongji Medical College, Huazhong University of Science and Technology, Wuhan 430000, China

**Keywords:** Heart failure, Cardiovascular disease, Global burden of diseases, Age-period-cohort model, Bayesian age-period-cohort model

## Abstract

**Background and Aims:**

Heart failure (HF) represents a major global health challenge. This study utilized the Global Burden of Disease (GBD) dataset to analyse HF epidemiology from 1990 to 2021 and project disease burden to 2035.

**Methods:**

We analysed GBD 2021 data on HF attributable to cardiovascular diseases (CVDs) for 1990–2021. Age-period-cohort modelling assessed the effects of age, time period, and birth cohort on HF. Joinpoint regression analysis characterized temporal trends and annual percentage changes (APC) in the age-standardized prevalence rate (ASPR) and years lived with disability (YLDs) for HF due to ischaemic heart disease (IHD) and hypertensive heart disease (HHD). The Bayesian age-period-cohort (BAPC) model projected HF patient numbers and ASPR for 2022–2035.

**Results:**

Globally, an increased risk of developing HF started at age 60 and after the period 2005–2009. Birth cohorts showed protective factors after 1955–1959. The Joinpoint model suggested that ASPR of HF resulting from IHD gradually decreased since 2019 (APC = −0.74, 95% UI: −0.94 to −0.55, *P* < .001). HHD showed a continuous upward trend in male patients, while decreasing in females after 2019 (APC = −0.47, 95% UI: −0.74 to −0.20, *P* < .01). Between 1990 and 2021, CVDs-attributable HF cases and YLDs rose from 20.1 million (95% UI: 17.3–23.4) and 1.92 million (95% UI: 1.28–2.66) to 45.6 million (95% UI: 39.7–52.7) and 4.32 million (95% UI: 2.96–5.94), respectively, representing increases of 127% and 126%. ASPR and age-standardized YLD rates increased by 3.74% and 3.36%. Projections indicate the global HF population will reach 27.0 million (95% UI: 21.6–32.5) males and 21.5 million (95% UI: 16.8–26.3) females by 2035, with stable ASPR.

**Conclusions:**

The rising absolute number of HF patients indicates a substantial CVD-attributable burden by 2035, necessitating enhanced focus on individuals over 60, particularly those with IHD and HHD.

## Graphical Abstract

**Graphical Abstract:**
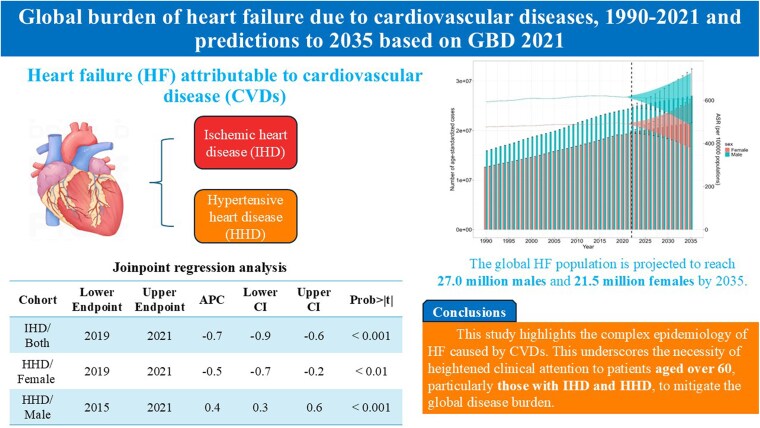

## Introduction

HF encompasses a range of syndromes characterized by impaired ventricular filling and/or ejection function, resulting in insufficient cardiac output, congestion of pulmonary and/or systemic circulation, and suboptimal organ and tissue perfusion. Major cardiovascular causes of HF include IHD, HHD, and rheumatic heart diseases, as well as arrhythmia, myocarditis, and alcoholic cardiomyopathy, with IHD being the leading cause of HF.^1^

As an advanced stage of CVDs, HF is associated with high mortality and rehospitalization rates. In developed countries, the age-adjusted incidence of HF is declining, however, due to the ageing population, the overall incidence is rising.^2,3^ The economic burden is substantial, with the total cost of HF in the USA projected to increase to US $69.8 billion by 2030.^4^ Currently, the incidence of HF in Europe is about 3/1000 person-years across all age-groups to 5/1000 person-years in adults,^5^ with a prevalence of approximately 1%–2% among adults.^6,7^ This prevalence may be underestimated, as studies often only include diagnosed cases.^5^ In China, the ASPR of HF is 0.57%, 3.86%, and 7.55% among people aged 25–64, 65–79, and ≥80 years, respectively.^8^ Regional variations in HF epidemiology reflect differences in the economic status, healthcare infrastructure, and healthcare policies.

In contrast to previous studies,^9^ this research provides a comprehensive analysis of global trends from 1990 to 2021 in the prevalence of HF attributable to cardiovascular diseases, as well as the corresponding YLDs. The analysis was stratified according to GBD super regions and socioeconomic development levels. Data obtained from the GBD outcomes tool were used to evaluate HF prevalence over this period and to forecast the future disease burden by estimating the number of HF cases anticipated by 2035.

## Methods

### Data sources

We used GBD 2021 data obtained from the Institute for Health Metrics and Evaluation (IHME), including 371 diseases and injuries across 204 countries and territories. All data were stored in the cloud to allow for free access through the GBD 2021 portal (https://vizhub.healthdata.org/gbd-results/). To extract the relevant information, we navigated to the ‘GBD Estimate’ category and selected ‘Impairment’, then chose ‘Heart Failure’ within that group. Under the ‘Cause’ section, we selected ‘Cardiovascular diseases’ and all associated subcategories. This study did not require ethical approval as it used publicly available data.

To assess HF, we focused on two key metrics: prevalence and YLDs. The data, which ranged from 1990 to 2021, was sourced from the Global Health Data Exchange (GHDx), and it included annual statistics on age, sex, and location. Our analysis encompassed 204 countries and territories, grouped into 21 GBD regions based on geographic proximity. Additionally, we used the sociodemographic index (SDI), a measure that quantifies sociodemographic development of a region based on income, education, and fertility circumstances.^10^

HF is classified as a Level 1 impairment in GBD 2021, clinically defined using validated diagnostic criteria—specifically the Framingham or European Society of Cardiology (ESC) guidelines. HF is further categorized into four clinical subgroups based on symptom severity: mild, moderate, severe, and treated HF (representing managed patients). Prevalence estimates align with American College of Cardiology/American Heart Association (ACC/AHA) stages C and D, which encompass symptomatic individuals with diagnosed HF, ranging from early symptomatic to end-stage disease.

### Age-period-cohort model analysis

The age-period-cohort model is an advanced research methodology with enhanced performance compared to the traditional analytical tools used in health and socio-economic development studies.^11^ The age-period-cohort model represents the overall and specific time trends, and estimates the impact of the fundamental time dimensions: age, period, and birth cohort.^12^

We utilized apc_ie to compute this constrained parameter vector by a special principal components regression. The IE uses the constraint that the sum of coefficients in each set is zero. After estimating the principal components regression, the IE uses the zero-sum constraints to obtain estimates for the deleted age, period, and cohort categories.^13^

We used the age-period-cohort model to explore the trends in HF prevalence across various age groups, time periods, and birth cohorts. This model differentiates the effects of age, period, and cohort to enhance the understanding of HF through various time periods and cohorts.

All analyses were performed using the apc_ie_case function in StataMP (Version 16). A Poisson regression model was specified with a log link function, and population exposure was incorporated as an offset variable to adjust for population size.

### Joinpoint regression analysis

We employed Joinpoint regression analysis using Joinpoint software (Version 4.9.1.0) to identify significant turning points in temporal trends related to global HF prevalence and YLDs attributable to CVDs. The analysis was further applied to examine the ASPR of HF and temporal patterns in YLDs resulting from IHD and HHD. Given the 32-year span of the data, a maximum of six joinpoints was permitted in the model.

### Prediction

In this study, we employed the Bayesian Age–Period–Cohort (BAPC) model to project future disease burden from 2022 to 2035, due to its ability to effectively handle the complex, high-dimensional, and often sparse data typical of large-scale epidemiological studies such as the GBD 2021. The BAPC model is built upon a generalized linear model (GLM) framework within a Bayesian setting, which allows for the dynamic integration of age, period, and cohort effects. These effects are modeled as time-varying and smoothed via a second-order random walk, improving the accuracy of posterior probability estimation. A major strength of the BAPC model is its use of the integrated nested Laplace approximation (INLA) method to efficiently approximate marginal posterior distributions.^14,15^

### Statistics

All statistical analyses and generation of graphs were performed using R Studio software (version 4.4.1) with R packages BAPC (version 0.0.36), INLA (version 24.06.27), ggplot2 (version 3.5.1), and StataMP (version 16), joinpoint (version 4.9.1.0). Al tests were two-tailed, with *P* < .05 considered statistically significant.

## Results

### Global trends

From 1990 to 2021, global HF prevalence experienced an absolute rise of 127%, with cases increasing from 20 105 008 (95% UI: 17 336 840–23 445 520) to 45 564 918 (95% UI: 39 741 018–52 704 642). ASPR increased by 3.74%, from 529.02 (95% UI: 456.46–620.56) to 548.81 (95% UI: 479.87–632.72) cases per 100 000 population. YLDs rose by 126%, with cases increasing from 1 915 140 (95% UI: 1 279 692–2 661 642) to 4 322 996 (95% UI: 2 957 608–5 943 710); the age-standardized YLDs rate increased by 3.36%, from 50.31 (95% UI: 33.70–69.97) to 52.00 (95% UI: 35.47–71.62) cases per 100 000 population. From 1990 to 2021, Australasia exhibited the largest estimated annual percentage change in ASPR of −0.48 (95% UI: −0.59 to −0.36), with the High-income Asia Pacific exhibiting the largest estimated annual percentage change in age-standardized YLDs with a value of −0.5 (95% UI: −0.62 to −0.38). The global ASPR and age-standardized YLDs were 0.14 (95% UI: 0.13–0.15) and 0.12 (95% UI: 0.1–0.13), respectively (Supplementary Table S1).

**Figure 1:**
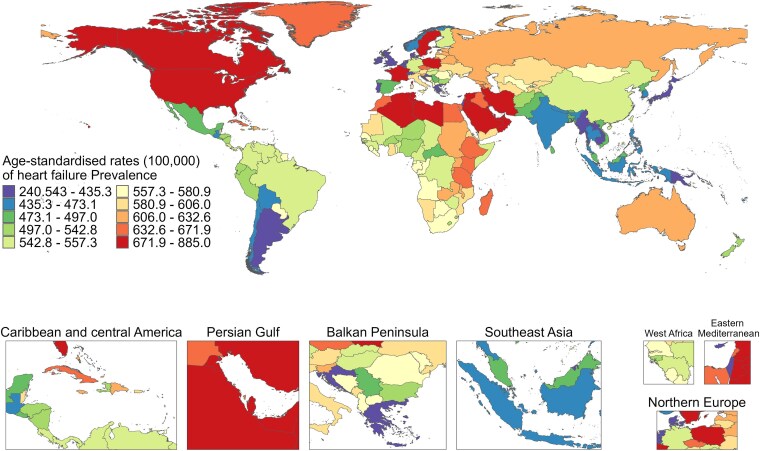
The global disease burden of HF for both sexes, showing ASPR across 204 countries and territories in 2021

**Figure 2:**
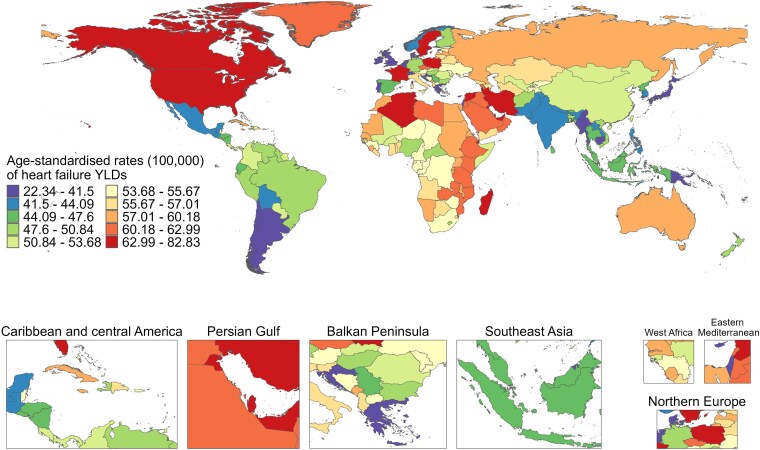
The global disease burden of HF for both sexes, showing age-standardized YLDs rate across 204 countries and territories in 2021

**Figure 3:**
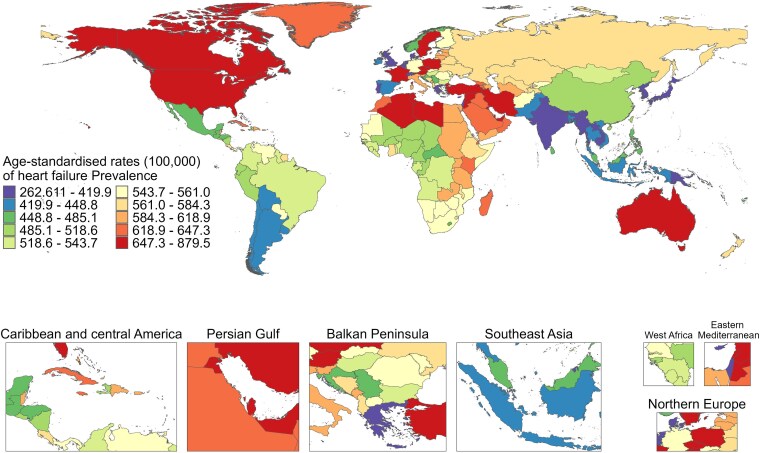
The global disease burden of HF for both sexes, showing ASPR across 204 countries and territories in 1990

**Figure 4:**
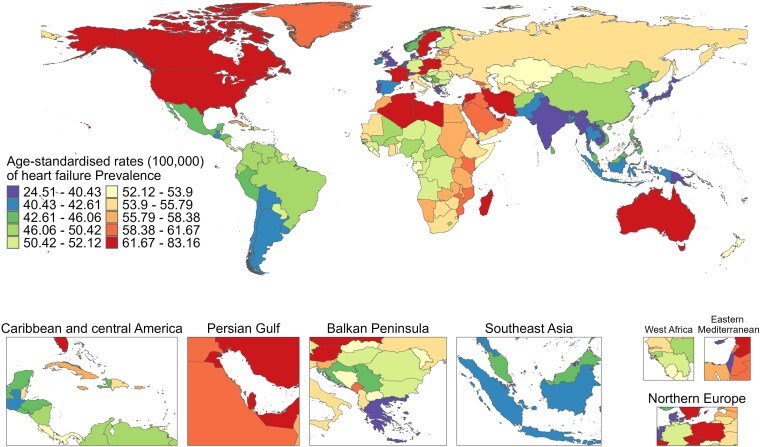
The global disease burden of HF for both sexes, showing age-standardized YLDs rate across 204 countries and territories in 1990

Among the 204 countries or regions, the ASPR was different between 1990 and 2021, with France (879.55, 95% UI: 723.19–1079.17), Sweden (866.23, 95% UI: 755.12–992.66), and Austria (859.23, 95% UI: 728.25–964.73) exhibiting the highest ASPR in 1990; whereas Sweden (884.95, 95% UI: 767.20–1029.58), France (881.96, 95% UI: 730.51–1065.39), and Poland (871.90, 95% UI: 807.29–939.55) with the highest ASPR in 2021 (*Figure 1*). The majority of the age-standardized rates of YLDs exhibited a similar trend to ASPR from 1990 to 2021 (*Figure 2*). *Figs 3 and 4* show the ASPR and age-standardized rates of YLDs in 1990.

### Overall temporal trends in gender and age structures

**Figure 5:**
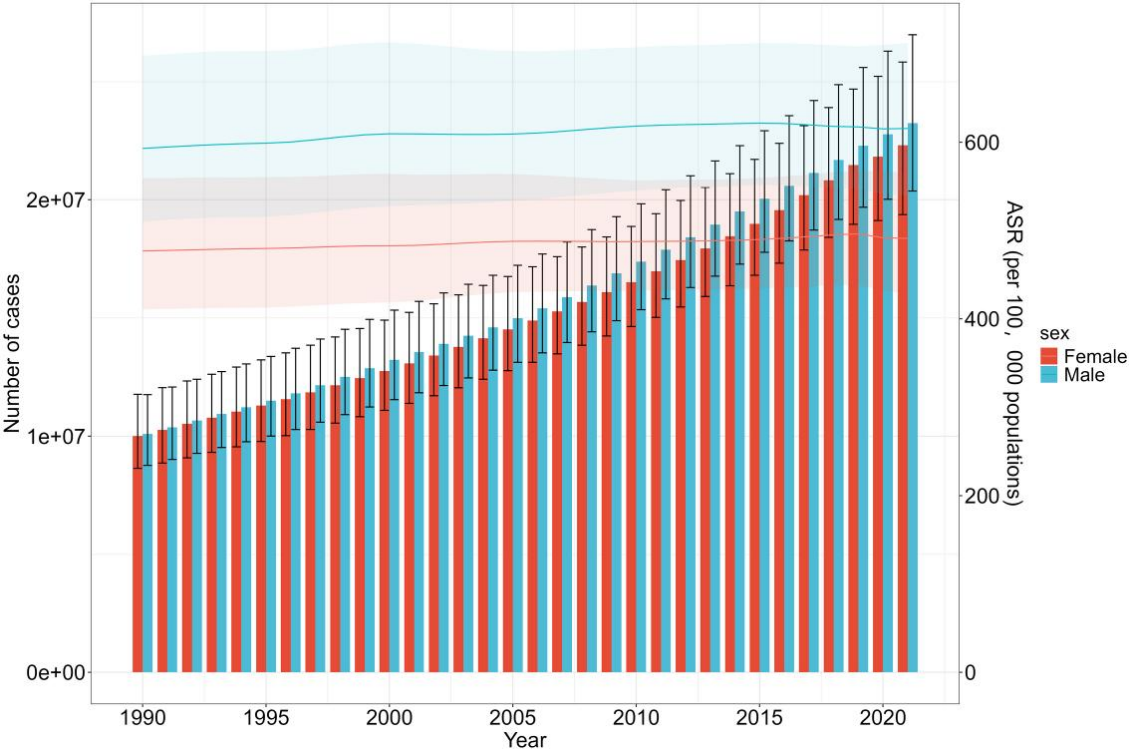
Global HF number of prevalent cases and ASPR between males and females

**Figure 6:**
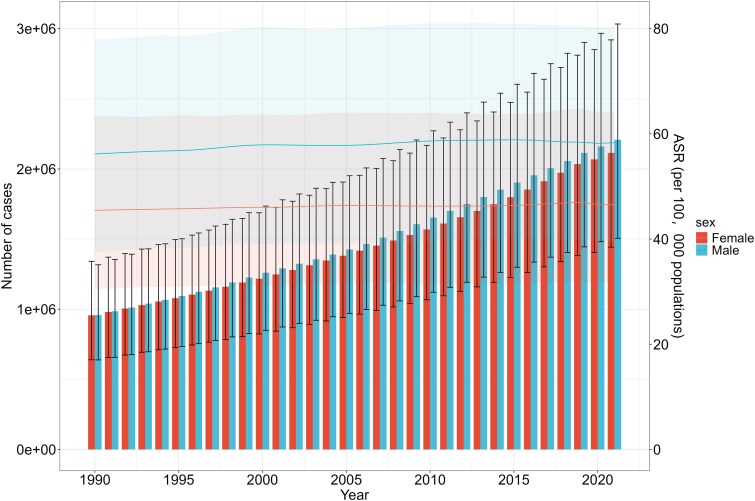
Global HF number of YLDs cases and age-standardized YLDs rate between male and female

From 1990 to 2021, the ASPR and age-standardized YLDs rates of HF showed no significant changes; however, the number of prevalent and YLDs cases across all age groups exhibited a gradual upward trend. Specifically, the ASPR and age-standardized YLDs rate were consistently higher in males compared to females during this period. The number of prevalent and YLDs cases across all age groups exhibited a rising trend with time (*Figs 5 and 6*).

### Contribution of risk factors to HF prevalence and YLDs

**Figure 7:**
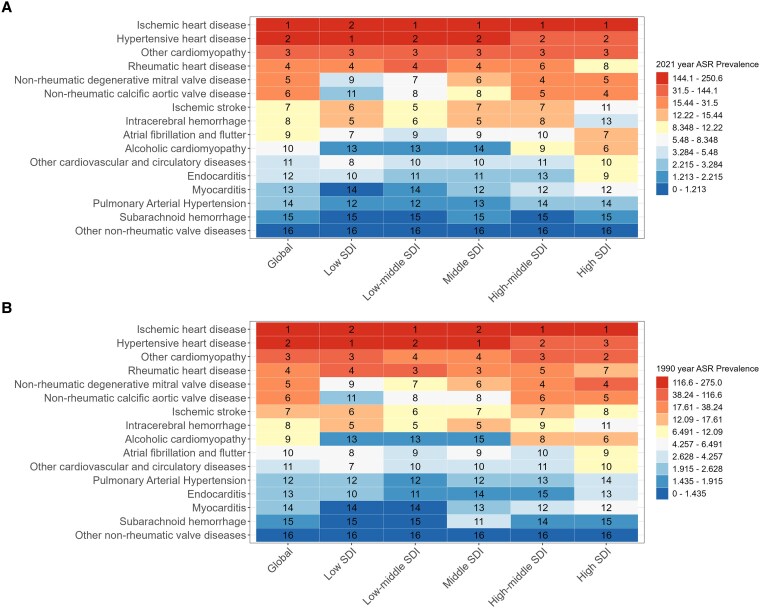
Ranking of ASPR HF prevalence attributable to risk factors globally and across the five SDI regions, for both sexes. (*A*) ASPR in 2021. (*B*) ASPR in 1990

**Figure 8:**
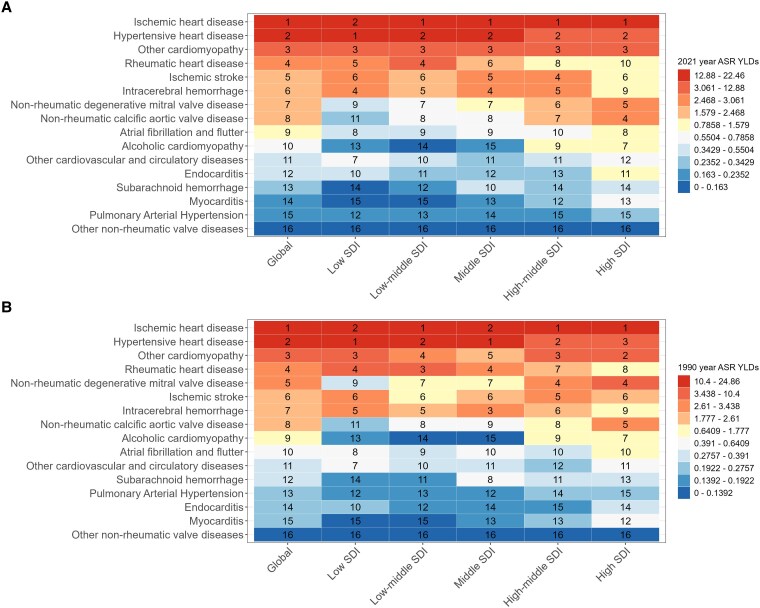
Ranking of age-standardized HF YLDs rates attributable to risk factors globally and across the five SDI regions for both sexes. (*A*) Age-standardized YLDs rate in 2021. (*B*) Age-standardized YLDs rate in 1990

Globally, the contribution of CVDs to the prevalence of HF and YLDs was approximately the same in 1990 and 2021. IHD and HHD contributed significantly, ranking first and second, respectively. Among the five SDI regions, 1990 and 2021, HHD was the leading risk factor for ASPR and age-standardized YLDs of HF in low Socio-Demographic Index (SDI) regions. In the middle SDI regions in 2021, IHD was the leading risk factor, followed by HHD, with the positions reversed for the conditions in 1990.(*Figs 7 and 8*).

### ASPR of HF and age-standardized YLDs rate trends from 1990 to 2021

**Figure 9:**
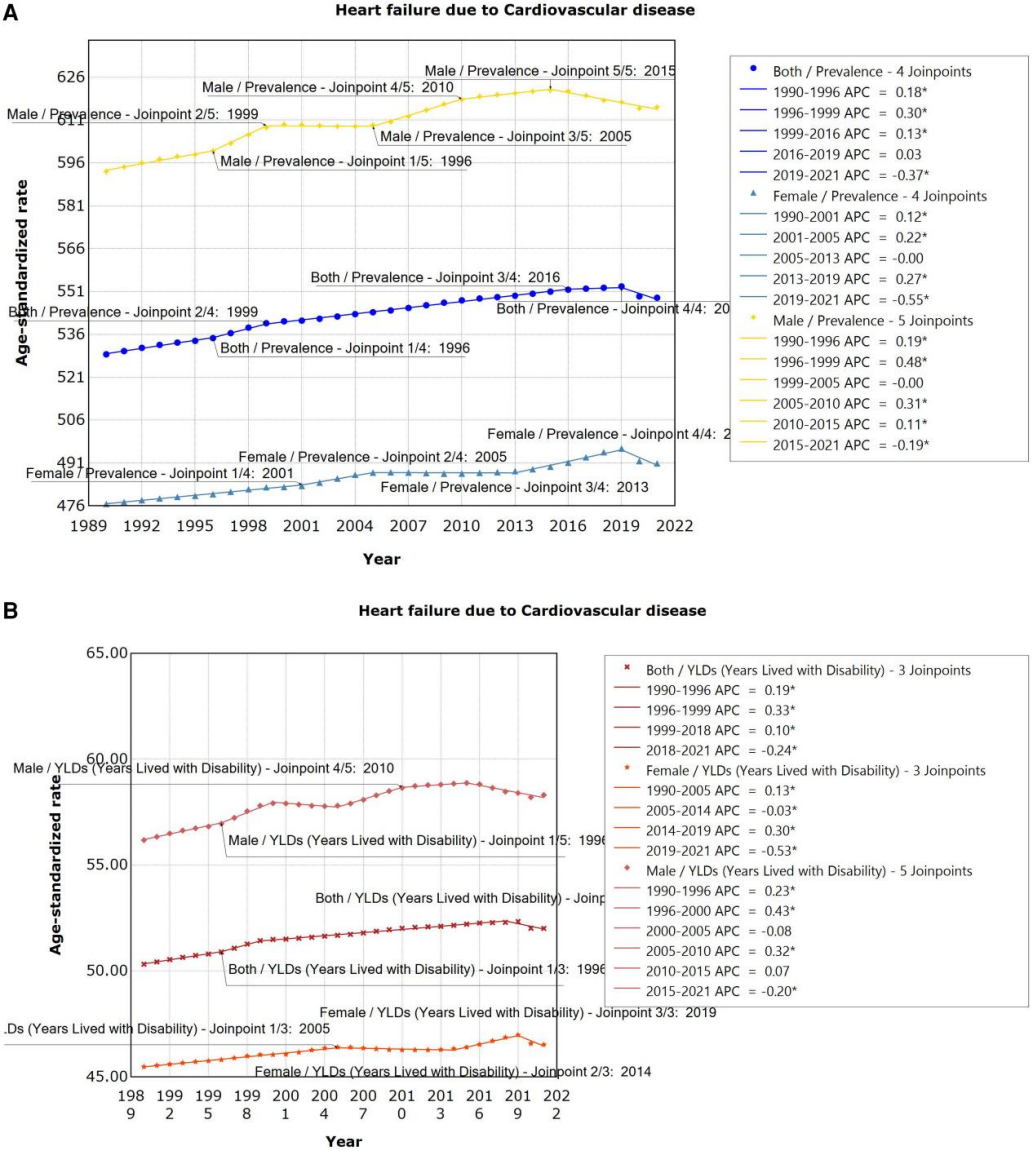
(*A*) The ASPR of HF due to CVDs. (*B*) The age-standardized YLDs rate of HF due to CVDs

*Figure 9A and B* illustrate trends in the ASPR of HF due to CVDs and age-standardized YLDs rate from 1990 to 2021. For ASPR, male, female, and both sexes showed an increasing trend from 1990 to 2019, peaking in 2019 before subsequently declining. Males exhibited the most pronounced increase from 1996 to 1999 (APC = 0.48, 95% UI: 0.26 to 0.70, *P* < .001), female from 2013 to 2019 (APC = 0.27, 95% UI: 0.24 to 0.30, *P* < .001), and both sexes from 1996 to 1999 (APC = 0.30, 95% UI: 0.16 to 0.44, *P* < .001). In terms of the age-standardized YLDs rates, males demonstrated varying increases before 2000, a decrease from 2000 to 2005, then continued to increase, peaking in 2015, and then declining. Females experienced a steady increase before 2005, a decrease from 2005 to 2014, then continued to increase, and subsequently declined after peaking in 2019. Both sexes demonstrated varying degrees of increase before 2018, which declined after the peak in 2018.

**Figure 10:**
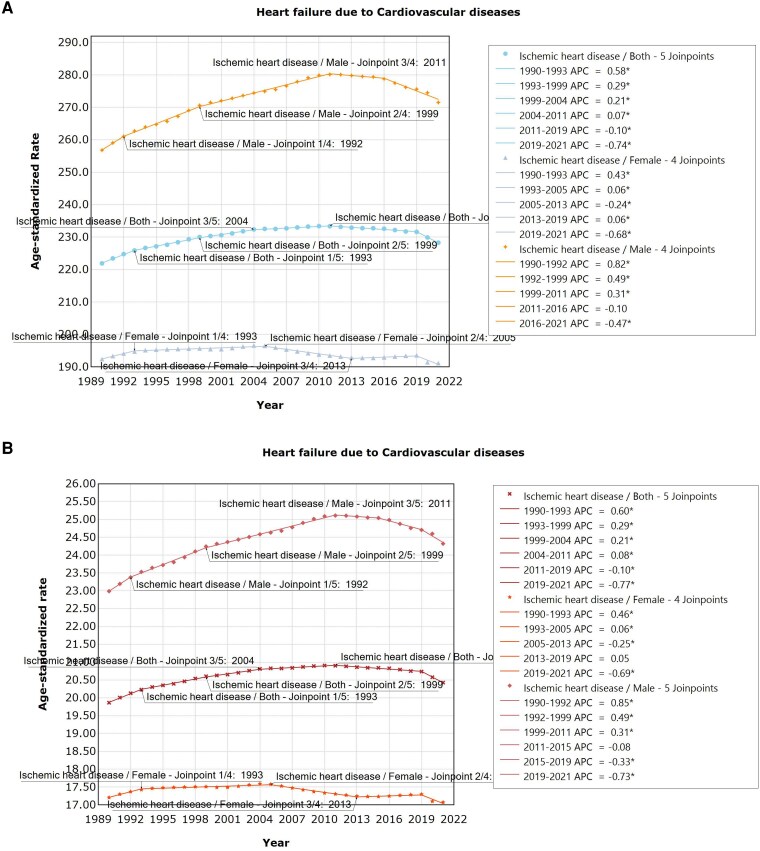
(*A*) The ASPR of HF due to IHD. (*B*) The age-standardized YLDs rate of HF due to IHD

In terms of HF caused by IHD, the overall prevalence and YLDs exhibited an increase, which was then followed by a decline. Specifically, men exhibited the highest prevalence between 1990 and 1992 (APC = 0.82, 95% UI: 0.23–1.41, *P* < .01), peaking in 2011, and then declining; for females, the prevalence was highest from 1990 to 1993 (APC = 0.43, 95% UI: 0.26–0.60, *P* < .001), peaking in 2005, followed by a decline from 2005, with a slight increased observed from 2013 to 2019, and a subsequent decline. For both sexes, the prevalence increased from 1990 to 1993 (APC = 0.58, 95% UI: 0.47–0.69, *P* < .001), reached the peak in 2011, and subsequently declined. The YLDs trend was similar to the prevalence (*Figure 10A and B*).

**Figure 11:**
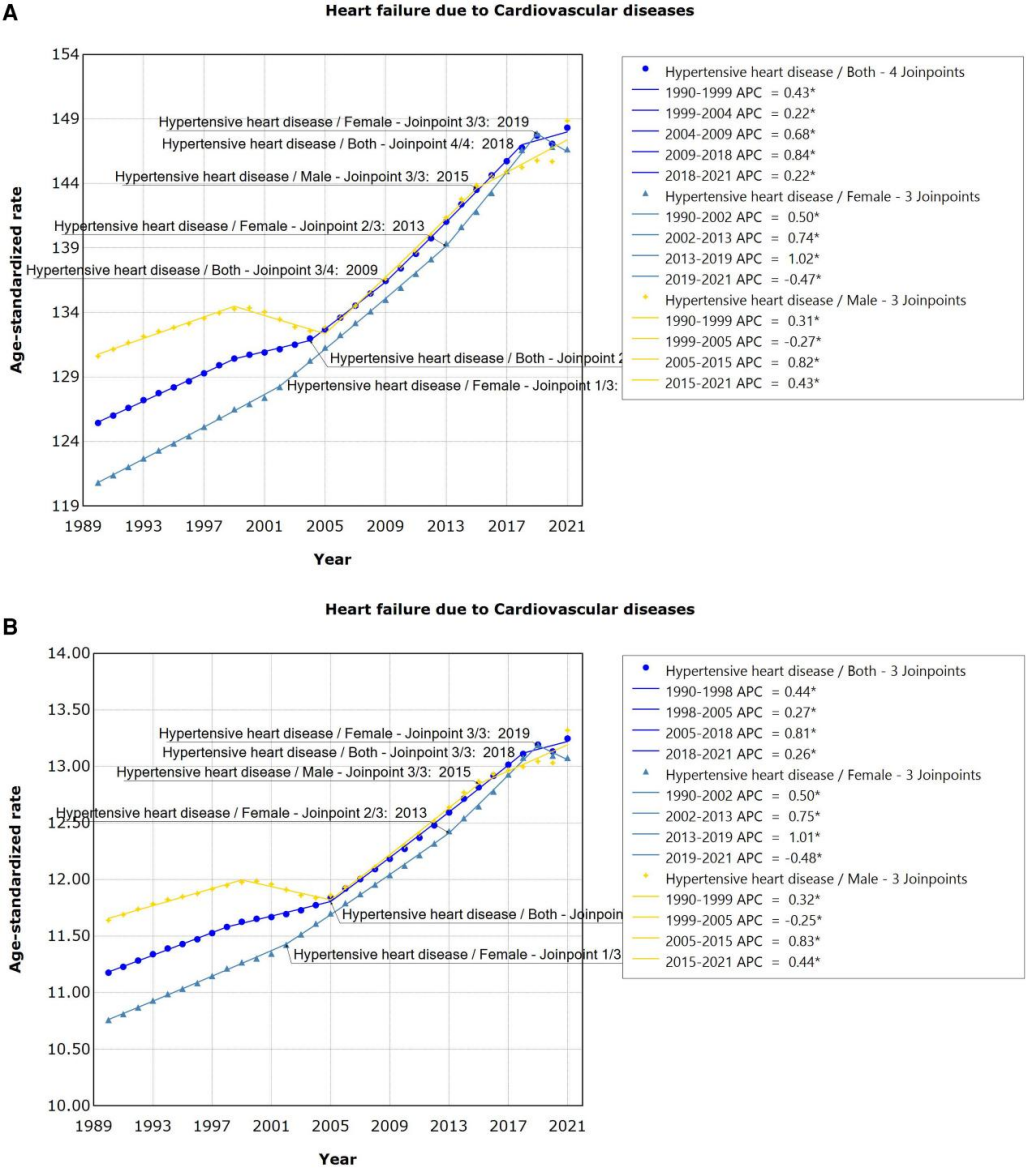
(*A*) The ASPR of HF due to HHD. (*B*) The age-standardized YLDs rate of HF due to HHD

For HF due to HHD, prevalence and YLDs exhibited a significant upward trend. Specifically, in men, the highest prevalence was recorded between 2005 and 2015 (APC = 0.82, 95% UI: 0.75–0.89, *P* < .001), which has been consistently rising. For females, the prevalence significantly rose from 2013 to 2019 (APC = 1.02, 95% UI: 0.96–1.07, *P* < .001), peaking in 2019, and subsequently declining. Both sexes exhibited increasing prevalence from 2009 to 2018 (APC = 0.84, 95% UI: 0.80–0.88, *P* < .001), with a continued rise since then. Similar to prevalence, trends in YLDs for HF due to HHD were observed (*Figure 11A and B*).

### Age, period, and birth cohort effects on the global prevalence of HF disorders

**Figure 12:**
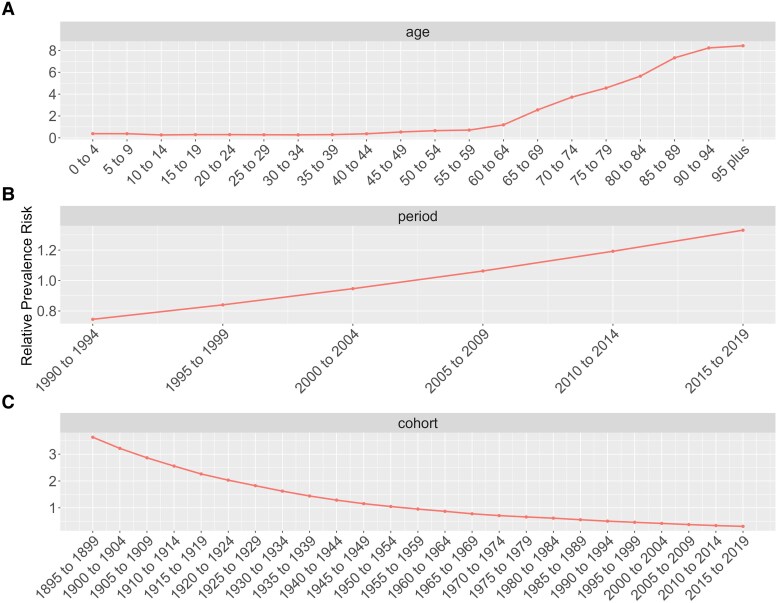
Age, period, and birth cohort effects on ASPR of HF globally, assessed using APC models. (*A*) The effects of age ASPR of HF were determined by adjusting for bias due to period and birth cohort. (*B*) Periods effects were determined by correcting for age and birth cohort factors. (*C*) Birth cohort effects were determined by adjusting for bias due to age and period

The age, period, and birth cohort effects of HF derived from the age-period-cohort model are shown in *Figure 12* and Supplementary Table S2. Age exhibited an increasing trend in relative risk, especially from 60 to 64 years, with its effects peaking at 95 years (*Figure 12A*).

The model indicated that over time, periods initially associated with favourable factors gradually transitioned into unfavourable ones. From 2005 to 2009, the age-related relative risk for HF shifted to unfavourable factors (*Figure 12B*). Globally, the birth cohort effect demonstrated a downward trend, indicating a gradual shift towards more favourable outcomes. Notably, cohorts born from 1955 to 1959 exhibited a significant transition in a positive direction (*Figure 12C*).

### Temporal trends in the sex and age distribution of individuals aged 60 years and older

**Figure 13:**
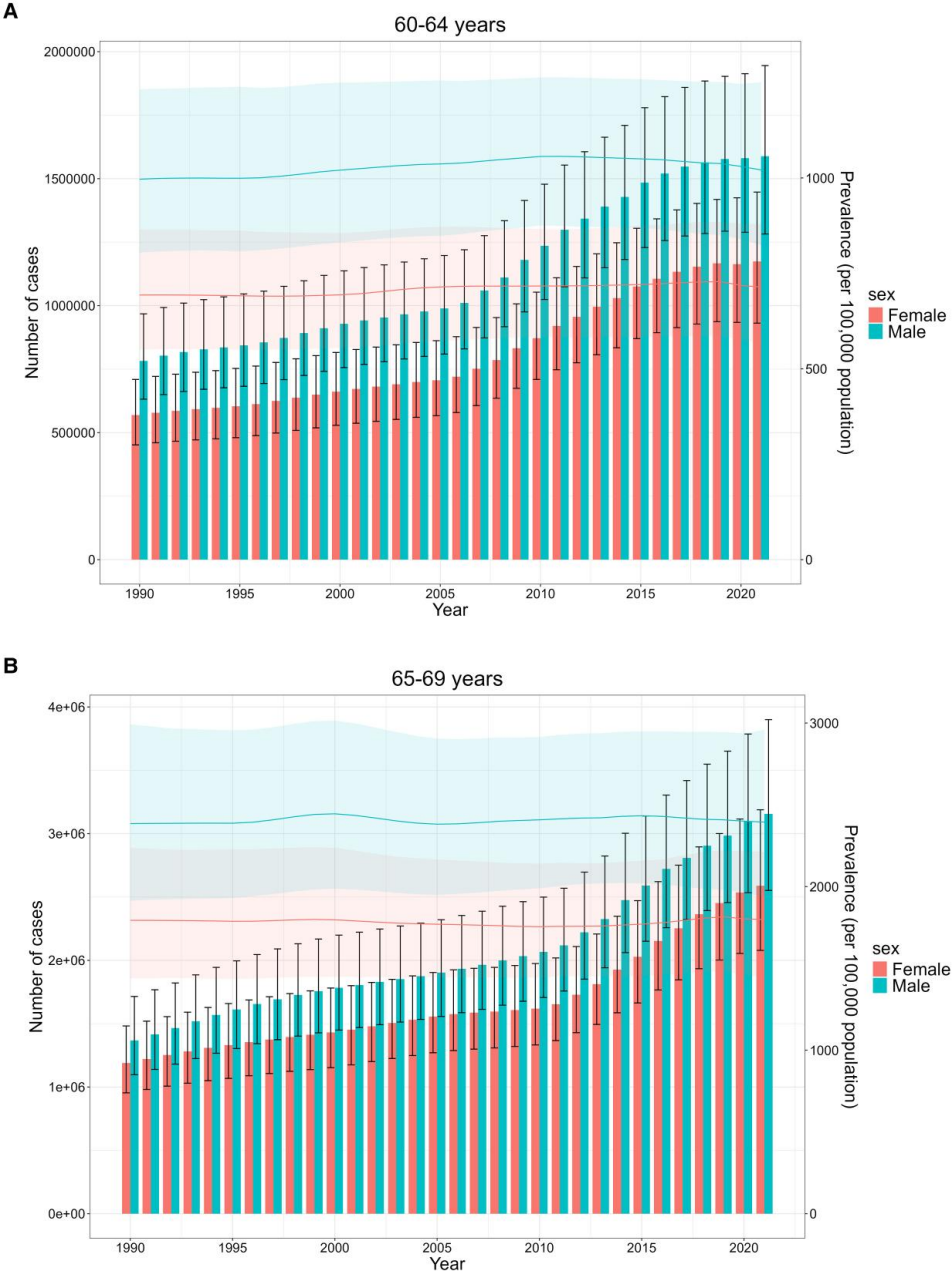

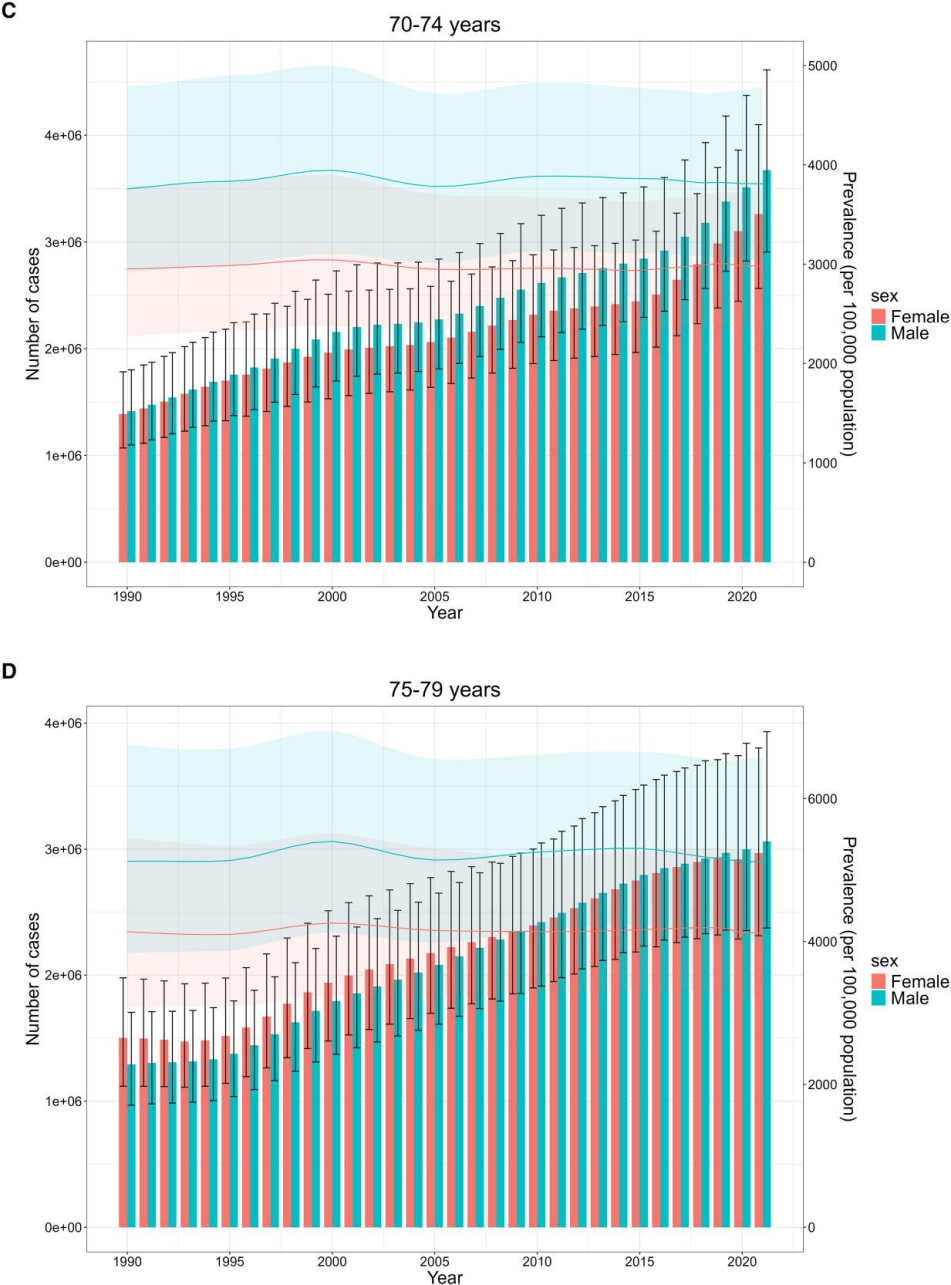

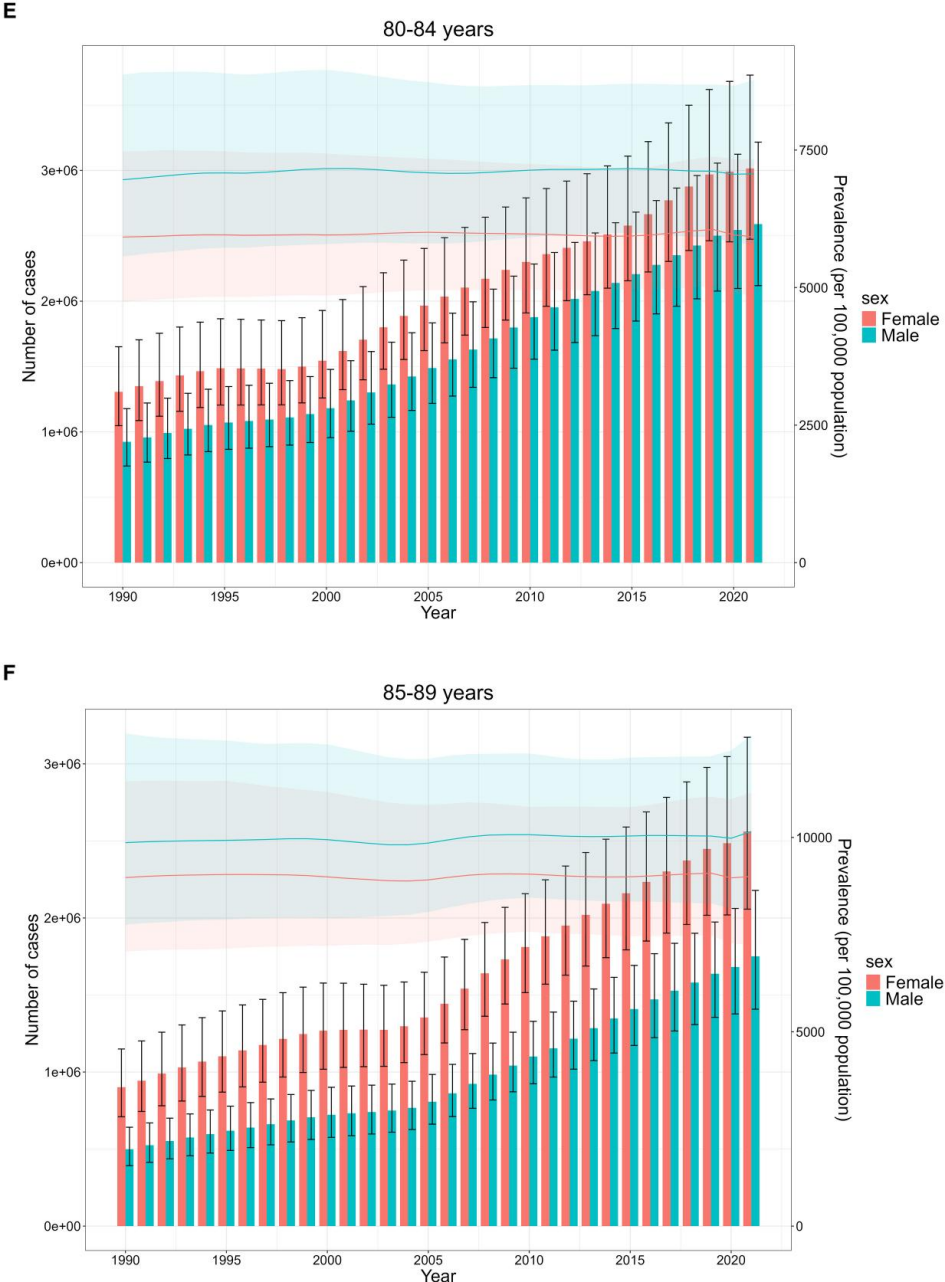

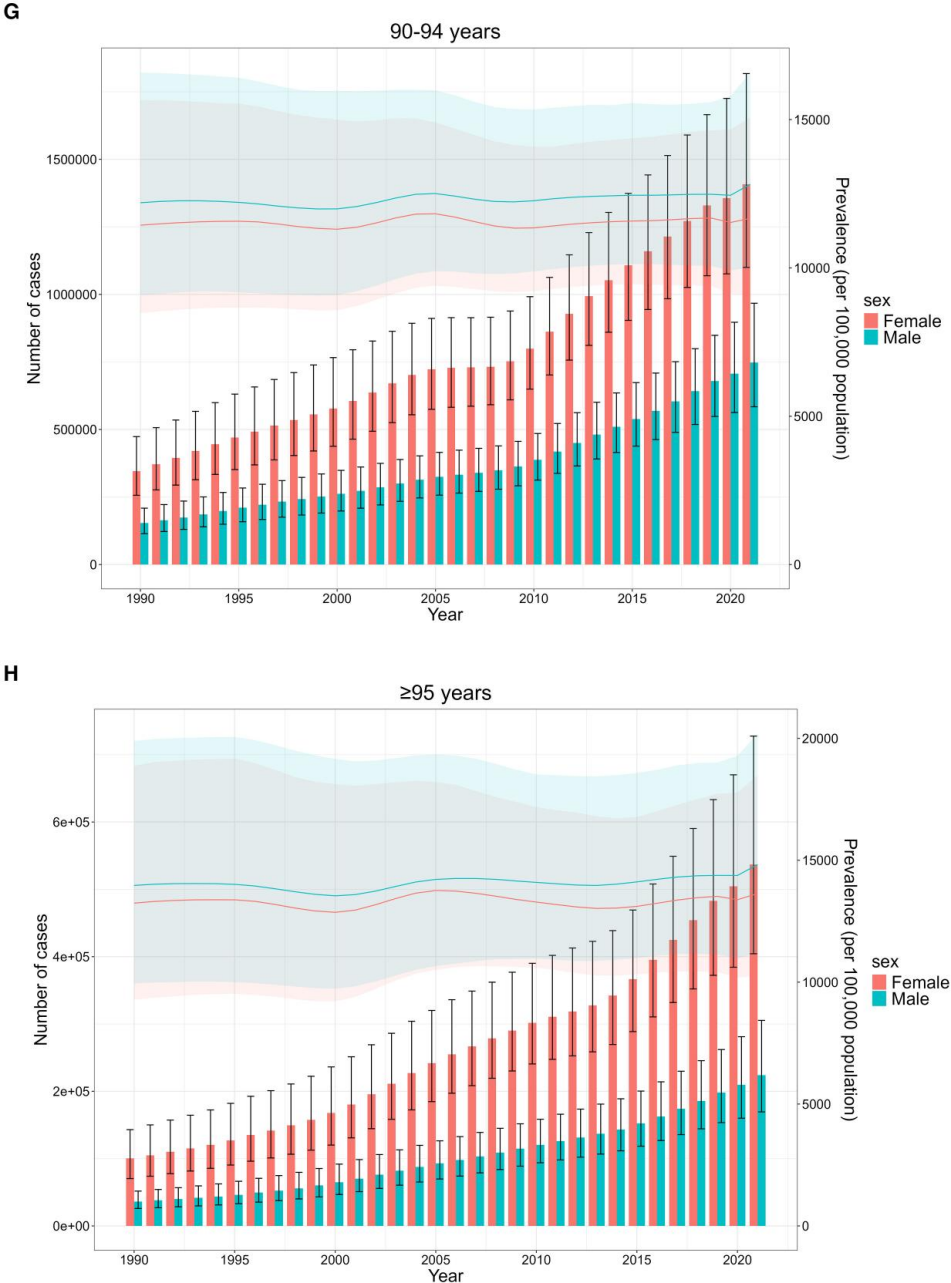
(*A*) Temporal trends in the number and prevalence rate of HF due to CVDs among adults aged 60–64 years. (*B*) Temporal trends in the number and prevalence rate of HF due to CVDs among adults aged 65–69 years. (*C*) Temporal trends in the number and prevalence rate of HF due to CVDs among adults aged 70–74 years. (*D*) Temporal trends in the number and prevalence rate of HF due to CVDs among adults aged 75–79 years. (*E*) Temporal trends in the number and prevalence rate of HF due to CVDs among adults aged 80–84 years. (*F*) Temporal trends in the number and prevalence rate of HF due to CVDs among adults aged 85–89 years. (*G*) Temporal trends in the number and prevalence rate of HF due to CVDs among adults aged 90–94 years. (*H*) Temporal trends in the number and prevalence rate of HF due to CVDs among adults aged ≥95 years

*Figure 13A–H* demonstrates the temporal trends in the number and prevalence of HF attributable to CVDs among individuals aged 60 years and older from 1990 to 2021. The number of male patients exceeded that of female patients in the age group of 60–74 years. In the 75–79 years age group, the number of female patients was significantly higher than that of male patients prior to 2009. In 2009, the number of male and female patients became comparable, after which the number of male patients began to exceed that of female patients. However, in the age group of 80–95 years and older, the number of female patients consistently remained higher than that of male patients. Regarding prevalence, men exhibited a consistently higher prevalence rate compared to women throughout the study period.

### The 2035 global disease burden prediction for HF

**Figure 14:**
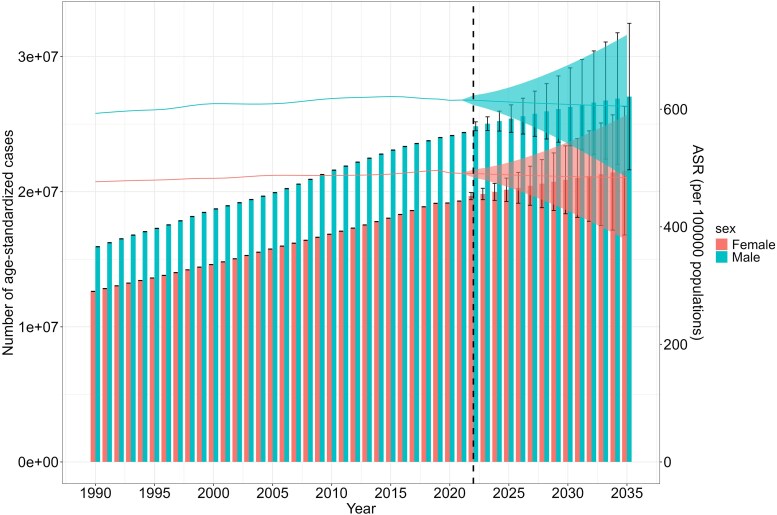
Projected ASPR and number of HF globally

We utilized the BAPC model to predict the trends in ASPR and the number of patients diagnosed with HF from 2022 to 2035. Globally, the number of men and women with HF is expected to continue rising from 2021 to 2035, reaching an estimated 27 037 154 (95% UI: 21 617 097–32 457 210) for men by 2035 and 21 547 981 (95% UI: 16 800 336–26 295 626) for women. Additionally, ASPR is expected to consistently reach 605.19 (95% UI: 483.87–726.51) for men in 2035, which is lower than the peak of 621.55 (95% UI: 621.27–621.83) in 2015. In women, the prevalence is projected to reach 483.82 (95% UI: 377.22–590.42) by 2035, lower than the peak of 496.10 (95% UI: 495.89–496.31) in 2019 (*Figure 14*).

## Discussion

This study systematically analysed the global, regional, and national epidemiological trends of HF attributable to CVDs from 1990 to 2021, with a focus on prevalence and YLDs. Despite substantial population growth during this period, the number of HF cases resulting from CVDs exhibited a sustained increase.^16^ Consequently, it is important to analyse the prevalence and YLD patterns of HF caused by CVDs, and to explore the underlying causes.

Globally, France exhibited the highest ASPR in 1990, followed by Sweden. Notably, the rankings of these two countries were reversed by 2021. It is worth mentioning that age-standardized YLDs during this period were approximately the same as ASPR. This phenomenon can be ascribed to the nation's advanced healthcare system, its accurate disease surveillance mechanisms, and the demographic structure characterized by a greater proportion of elderly residents. From 1990 to 2021, the number of male patients, as well as the ASPR and YLDs, were consistently higher than those observed in females. The difference between male and female is associated with the protection provided by oestrogen on the cardiovascular system.^17,18^

The age-period-cohort model analysis revealed a consistent age-dependent escalation in HF ASPR beginning at 60–64 years, which aligns with findings from other global burden studies on HF.^16,19^ Numerous studies have shown that age is an important risk factor for HF, with the incidence of HF doubling per decade in men and tripling in women after 65 years of age.^20,21^ With ageing, biological ageing caused by mitochondrial dysfunction and decreased protein regulation function leads to left ventricular hypertrophy, reduced left ventricular diastolic function, atrial fibrillation, and myocardial fibrosis, which make the elderly more susceptible to HF.^22,23^ Subgroup analysis of HF patients aged ≥60 indicated that male patients outnumbered female patients between 60 and 74 years. In the 75–79 age group, female patients significantly exceeded male patients prior to 2009; however, this trend reversed after 2009, with male patients gradually surpassing female patients. Notably, in the 80–95 plus age group, female patients consistently outnumbered male patients, potentially due to delayed onset and longer survival in women compared to men.^24,25^ In the period effect model, the period from 2005 to 2009 was an adverse factor, which may be related to the progress in HF diagnosis technology and environmental changes.^26,27^ With the widespread adoption of NT-Pro BNP and BNP testing, along with the implementation of comprehensive diagnostic and treatment strategies for HF, an increasing number of individuals have been diagnosed at earlier stages of the disease. It makes that the growing global population has been linked to a higher incidence of HF.^28,29^ The birth cohort analysis indicated a shift towards a protective effect beginning with the 1955–1959 cohort, likely attributable to advancements in medical technology and the expansion of healthcare services. Technological advancements have enabled more patients to receive early standardized diagnosis and treatment, which prevents exacerbation of the disease.^2,30^

For the HF caused by CVDs, IHD and HHD ranked highest for ASPR and age-standardized YLDs globally in 1990 and 2021.^31^ Across the five SDI regions, the causes of HF in 1990 and 2021 were slightly different. In 1990, HHD ranked first in low SDI and middle SDI regions, followed by IHD. In 2021, the causes of HHD in low SDI areas were similar to those in 1990, as well as in other regions and globally. The joinpoint model demonstrated that ASPR and age-standardized YLDs of HF caused by IHD declined after 2019, which was associated with the development of interventional technology, standardized treatment of HF, and improved awareness of HF.^32,33^ ASPR and age-standardized YLDs of HF caused by HHD are continuously increasing, this phenomenon is associated with poor eating habits and obesity.^34,35^ Women exhibited a downward trend after 2019, which might be attributed to their increased focus on weight management and body composition. Studies have indicated that ischaemic HF due to heart disease exhibits sex-specific phenotypic divergence: HFpEF (HF with preserved ejection fraction) is more prevalent among female patients, whereas HFrEF (HF with reduced ejection fraction) predominates in males. This discrepancy may be attributable to men suffer more frequently from occlusive epicardial CAD, whereas women exhibit more frequently a non-obstructive CAD or endothelial/microvascular dysfunction.^25,36^ HHD predominantly drives the development of HFpEF. Studies have demonstrated that hypertension induces alterations in myofilament protein stiffness and promotes myocardial fibrosis, which collectively impair diastolic function. Over time, these pathological changes may progress to systolic dysfunction, ultimately contributing to HFpEF.^37–39^

Although the absolute number of heart failure cases due to CVDs has continued to grow for over thirty years, the ASPR has shown a plateauing trend. This apparent contradiction can likely be explained by a substantial increase in the general population during the same period, which dilutes the proportional impact when rates are age-standardized.^16^ Using the BAPC model, it is projected that by 2035, the number of men with HF due to CVDs will reach 27 037 154 and 21 547 981 for women, with the ASPR being 605.19 (per 100 000) for men and 483.82 (per 100 000) for women. HF primarily affects the elderly, due to the decline of physical and metabolic functionality, thereby significantly increasing the risk of CVDs.^40–42^

As the global population ages, the steady increase in HF cases poses a significant challenge for the development of healthcare strategies and health service policies. Firstly, clinicians can enhance the early detection of HF by measuring natriuretic peptides, thereby improving patient outcomes and reducing healthcare expenditures.^43,44^ Secondly, the global longitudinal strain derived from echocardiography can serve as a valuable tool for assessing the prognosis of HF.^45,46^ Additionally, the evaluation of left atrial (LA) compliance (ratio of LA reservoir strain to E/e’) during exercise compared to resting conditions or exercise E/e’ ratio alone, may assist in diagnosing HFpEF.^47^ In the management of HF, patients diagnosed with HFrEF should be treated with the four pillars for HF treatment, which include beta-blockers, angiotensin-converting enzyme inhibitors (ACEIs)/angiotensin receptor-neprilysin inhibitors (ARNI) such as sacubitril/valsartan or angiotensin receptor blockers (ARBs), mineralocorticoid receptor antagonists (MRAs), and sodium-glucose cotransporter-2 (SGLT2) inhibitors.^45^ For patients with HFpEF, SGLT2 inhibitors, ARNIs, and MRAs should be administered based on their specific clinical characteristics.^48^ Additionally, for discharged patients, regular follow-up is recommended to facilitate the adjustment of pharmacological therapy and promote exercise rehabilitation and recovery of cardiopulmonary function.^49^ Given that HF patients often present with comorbidities such as hypertension, diabetes, obesity, and chronic kidney disease, it is essential to identify treatable comorbid conditions and develop systematic treatment strategies that incorporate the management and control of these concurrent diseases.^50^

To tackle the growing burden of HF, public health strategies must prioritize implementing effective prevention and control measures.

First, priority should be given to the reallocation of healthcare resources to mitigate existing disparities in medical service access. Studies have shown that between 2019 and 2021, the age-standardized prevalence of heart failure declined significantly in high-SDI regions, while it continued to increase in other areas.^19^ This disparity underscores the need for further attention, and future policies must be designed to foster a more equitable global distribution of healthcare resources, thereby ensuring balanced access across all regions.

Second, high-risk individuals for heart failure—especially those with comorbid metabolic disorders including hypertension, hyperlipidaemia, hyperglycaemia, and obesity—should be prioritized for systematic monitoring. Community and primary care providers should perform regular follow-ups to evaluate the management of blood pressure, lipids, glucose, and body weight, and implement timely interventions when necessary.

Third, individuals diagnosed with pre-heart failure (Stage A), preclinical heart failure (Stage B), or those identified as high-risk should receive regular cardiac evaluation—including echocardiography and B-type natriuretic peptide (BNP) testing—to assess cardiac structure and functional status.^51^ Timely and targeted intervention in these populations is crucial to prevent or delay the progression to overt heart failure.

Fourth, in patients hospitalized with heart failure, specialists should prioritize early mobilization.^52^ Before discharge, comprehensive disease management education, individualized medication regimens, and personalized guidance on cardiac rehabilitation should be provided. These measures are anticipated to lower readmission rates, improve long-term outcomes, and enhance overall quality of life. Furthermore, leveraging advances in artificial intelligence, wearable devices can enable continuous remote monitoring of patients after discharge, facilitating timely interventions based on real-time health data to support recovery.^53–55^

Finally, media outlets and community organizations should engage in strategic collaboration to promote cardioprotective lifestyles among the broader population. Key initiatives should include advocating regular moderate-intensity physical activity, maintenance of a healthy body weight, and avoidance of smoking and excessive alcohol consumption, with the goal of improving cardiovascular health at the population level.

### Limitations

Although this study provides valuable insights into clinical practice by analysing global HF trends, it is not without limitations.

First, the data utilized in this study were sourced from various countries and submitted to the GBD collaboration group, which may introduce variability in data quality.

Second, due to data constraints, the analysis was limited to an overall assessment of HF, precluding subgroup analyses for HFrEF, HFmrEF, and HFpEF.

Third, the assumptions employed to evaluate the causes of HF in the GBD data are constrained by the presumption of a single cause of HF, thereby failing to account for multifactorial aetiologies.

Fourth, an important consideration in interpreting the observed trends is the evolution of diagnostic criteria over time. As the understanding of heart failure has advanced, diagnostic standards have been periodically updated. In addition, continuous improvements in diagnostic tools and techniques may have enhanced the accuracy and earliness of detection, potentially increasing reported prevalence rates irrespective of true changes in disease incidence.

Fifth, while the GBD 2021 study integrates a broad range of data sources, limitations remain in the availability and completeness—particularly in low- and middle-income countries and for certain historical periods. Furthermore, heart failure estimates in the GBD framework are partly derived through statistical modelling rather than direct empirical measurement in all regions, which may affect accuracy in under-represented populations. These methodological aspects should be carefully considered when interpreting the results.

Finally, the analysis focused solely on CVDs as a whole and on HF attributable to IHD and HHD, the most prevalent causes, without detailed examination of other potential causes.

## Conclusion

This study explored the changing global burden of HF and emphasized the impact of age, period, and birth cohort on the ASPR of HF due to CVDs. The study analyses the impact of ischaemic and HHD on HF, projects the trend in the number of patients with HF attributable to CVDs, and the ASPR of HF from 2022 to 2035. Consequently, we conclude as follows: increasing efforts should be directed towards the elderly over 60 years and patients with IHD or HHD, since they are at a high risk of HF. To mitigate the global burden of HF, tailored programmes are needed for both the general public—including health education, exercise, and diet guidance, smoking cessation, and management of blood pressure, glucose, and lipids—as well as for discharged patients, who require dedicated rehabilitation guidance and systematic follow-up.

## Supplementary Material

xvag038_Supplementary_Data

## Data Availability

All data were store in the cloud to allow for free access through the GBD 2021 portal (https://vizhub.healthdata.org/gbd-results/).
